# Altered brain metabolic connectivity at multiscale level in early Parkinson’s disease

**DOI:** 10.1038/s41598-017-04102-z

**Published:** 2017-06-26

**Authors:** Arianna Sala, Silvia Paola Caminiti, Luca Presotto, Enrico Premi, Andrea Pilotto, Rosanna Turrone, Maura Cosseddu, Antonella Alberici, Barbara Paghera, Barbara Borroni, Alessandro Padovani, Daniela Perani

**Affiliations:** 1 Vita-Salute San Raffaele University, Via Olgettina, 58, 20132 Milan, Italy; 2 Division of Neuroscience, San Raffaele Scientific Institute, Via Olgettina, 58, 20132 Milan, Italy; 3 Neurology Unit, Department of Clinical and Experimental Sciences, University of Brescia, piazzale Spedali Civili, 25123 Brescia, Italy; 4 Parkinson’s disease Rehabilitation Centre, FERB ONLUS S. Isidoro Hospital, Trescore, Balneario Italy; 5 Nuclear Medicine Unit, Azienda Ospedaliera “Spedali Civili”, Spedali Civili Hospital, 25123 Brescia, Italy; 6 Nuclear Medicine Unit, San Raffaele Hospital, Via Olgettina, 60, 20132 Milan, Italy

## Abstract

To explore the effects of PD pathology on brain connectivity, we characterized with an emergent computational approach the brain metabolic connectome using [18F]FDG-PET in early idiopathic PD patients. We applied whole-brain and pathology-based connectivity analyses, using sparse-inverse covariance estimation in thirty-four cognitively normal PD cases and thirty-four age-matched healthy subjects for comparisons. Further, we assessed high-order resting state networks by interregional correlation analysis. Whole-brain analysis revealed altered metabolic connectivity in PD, with local decreases in frontolateral cortex and cerebellum and increases in the basal ganglia. Widespread long-distance decreases were present within the frontolateral cortex as opposed to connectivity increases in posterior cortical regions, all suggestive of a global-scale connectivity reconfiguration. The pathology-based analyses revealed significant connectivity impairment in the nigrostriatal dopaminergic pathway and in the regions early affected by α-synuclein pathology. Notably, significant connectivity changes were present in several resting state networks especially in frontal regions. These findings expand previous imaging evidence of altered connectivity in cognitively stable PD patients by showing pathology-based connectivity changes and disease-specific metabolic architecture reconfiguration at multiple scale levels, from the earliest PD phases. These alterations go well beyond the known striato-cortical connectivity derangement supporting *in vivo* an extended neural vulnerability in the PD synucleinopathy.

## Introduction

Parkinson’s disease (PD) is a neurodegenerative disease predominantly characterized by abnormal intracellular accumulations of insoluble α-synuclein into fibrils^1^. It has been postulated that the synaptic dysfunction caused by the small aggregates of α-synuclein is the initial event leading to neurodegeneration in PD and in other synucleinopathies^2^. Since physiological α-synuclein plays a key role in the regulation of the dopaminergic normal synaptic function, dopaminergic neurons are particularly vulnerable to α-synuclein pathology^2^. Synaptic dysfunction might impair both neurotransmitter release and regulation of synaptic plasticity mechanisms, also producing widespread effects on functional connectivity among distant brain regions which may result in neural networks alterations^3^. All above evidence suggests that PD can be regarded as a “network-opathy”^4^, and that adopting a network perspective is essential to understand its pathophysiology^5^. During the last years, an increasing number of neuroimaging studies reported networks alterations in the PD brain. The most consistently reported connectivity change is the alteration of the striato-cortical loop, both in the form of microstructural damage, as shown by diffusion tensor imaging, or as functional connectivity changes by resting state fMRI (rs-fMRI (see refs 5 and 6). Connectivity changes between striatal and cortical regions have been associated with resting tremor^7^, freezing of gait^8^ and overall motor symptoms severity, as measured by UPDRS-III score^9^, thus suggesting that impairment of the cortico-striatal loops is a key phenomenon for the occurrence of motor symptoms in PD^6^. A limited amount of rs-fMRI studies assessed connectivity in non-motor resting state networks, reporting reduced connectivity in (i) default mode network (DMN), correlating with working memory and visuo-spatial scores^10^, (ii) attentional network, also associated with presence of visual hallucinations^11^ and (iii) fronto-parietal network, correlating with executive performances^12^. Altogether, these studies show that several changes in connectivity underlie the symptomatic aspects of PD^5^, and that the dopaminergic degeneration inducing loss of striato–cortical functional connectivity is only part of a larger picture^9^. Notably, as recently suggested, not the dopaminergic cell death, but the abnormal accumulation of α-synuclein could be the initial event leading to neurodegeneration in PD and in other synucleinopathies^2^. α-synuclein aggregation affects key synaptic proteins, impairing neuronal function and axonal transport^1, 2^. These effects on both neurotransmitter release and regulation of synaptic mechanisms can affect activity-dependent signalling and gene expression^3^ leading to synaptic disconnection and network disintegration^3, 5^.

A unique tool to capture *in vivo* the pathological events that contribute to synaptic dysfunction is [18F]fluorodeoxyglucose with positron emission tomography ([18F]FDG-PET). [18F]FDG-PET is considered as an effective measure of energy consumption in neurons (specifically in synapses)^13^, and its signal has also been associated with synaptic density and function^14^, altered intracellular signalling cascades, impaired neurotransmitter release, spreading of proteinopathies, and long distance disconnection (see ref. 15). Crucially, brain energy consumption as measured by [18F]FDG-PET reflects neuronal communication signalling^16^, and it is thus strictly interrelated with functional connectivity^17, 18^. Thus, a metabolic network perspective might allow for important insights into local neural vulnerabilities, long-range disconnection, and the effects of neuropathology. In the last years, several approaches have been developed in order to assess metabolic connectivity in the human brain^19, 20^. Based on the assumption that regions whose metabolism is correlated are functionally interconnected^21^, seed-based voxel wise analysis^20^ and Sparse Inverse Covariance Estimation (SICE) method^19^ represent suitable approaches to measure functional interconnections between brain regions. These methods provide results comparable to those derived by rs-fMRI images analyses^22^, allowing also the application of graph-theoretical analysis to [18F]FDG-PET data^19^. SICE method is particularly suitable for the assessment of [18F]FDG-PET brain metabolic connectivity providing reliable estimation even with relatively small sample sizes. This is extremely useful for [18F]FDG-PET connectivity studies in which a single mean image is obtained for each subject and the sample size corresponds to the number of subjects and not to the length of the time series, as in rs-fMRI studies^19^.

The SICE method has been previously applied to [18F]FDG-PET data in neurodegenerative diseases for connectome assessment^19, 23, 24^. Notably, in our previous work^24^, we provided a metabolic connectome assessment for dementia with Lewy bodies (DLB), a neurological condition belonging to the α-synuclein spectrum, as PD. To the extent that metabolic connectivity captures the underlying synaptic dysfunction and pathology, the assessment of metabolic connectivity in diseases belonging to same neuropathological spectrum might reveal similar patterns of neural vulnerability. Since connectivity could be a marker of the clinical phenotype^25^, disease-specific patterns of connectivity changes need to be explored. Metabolic connectivity signatures indeed, might provide a pathology-based and clinically-related endophenotype, allowing a better understanding of differences and consistencies in diseases sharing the same underlying pathology but presenting with different clinical phenotypes.

With the aim to further explore brain metabolic connectivity in the synucleinopathies spectrum, we applied both seed-based and SICE approaches for metabolic connectivity estimation at multiple scale levels in a cohort of PD cognitive stable patients. We considered (i) local and long-distance connectivity in the whole brain, (ii) the dorsal and ventral dopaminergic pathways^26^, (iii) the spreading of α-synuclein pathology, according to Braak’s stages^27^. In addition, we assessed the possible dysfunctional changes in the high-order resting state networks, namely the attentional, anterior and posterior DMN, executive and motor networks in the same PD patients.

## Results

### Whole-brain analysis

#### Number of connections

**Figure 1:**
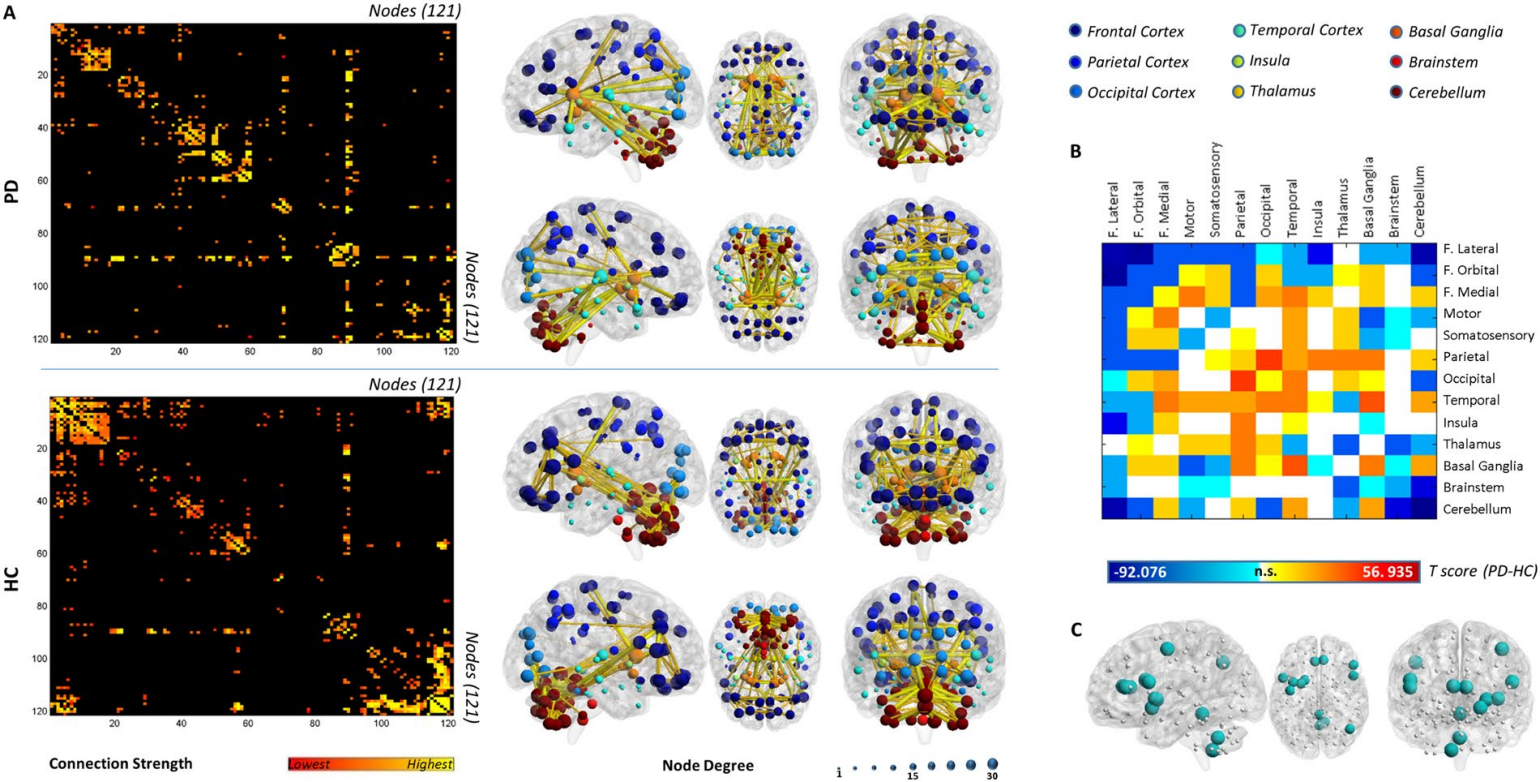
Whole-brain analysis. (**A**) Figure shows the whole-brain connectivity matrices of PD and HC (first column), with the red-yellow colour gradient representing the strength of the correlation between two nodes. The second column shows PD and HC connectomes projected on a 3D brain template. A global connectivity reconfiguration is present, with frontal and cerebellar regions being the most affected in PD patients. Node degree, i.e. the number of connections for each region, is represented by the size of each dot. Connection strength is represented by the colour and thickness of each line connecting two nodes. (**B**) The T-score matrix shows differences in the number of connections within and between each sub-matrix in PD versus HC. Abbreviations: F: Frontal. (**C**) Figure shows lost hub regions, i.e. nodes that assume the role of hubs in HC but not in PD. Hub identification was based on participation coefficient. Hubs are represented on a 3D brain template using BrainNet toolbox.

PD metabolic connectivity matrix showed a whole-brain reconfiguration with a prevalent loss of connectivity in frontal and cerebellar structures (Fig. 1A). Statistical testing of the differences in the number of connections in each submatrix across groups revealed significant *local connectivity decreases* within a number of sub-matrices, namely the frontolateral, orbitofrontal, occipital, thalamic, brainstem and cerebellar submatrices. The connectivity impairment in frontolateral and cerebellar submatrices was the most severe. Significant *local connectivity increases* were found instead in the frontal medial, parietal, temporal and basal ganglia submatrices (Fig. 1B).

As for the long-distance connectivity changes, a pattern of widespread long-distance disconnections was found involving the submatrices whose metabolic connectivity was locally decreased as well, namely the frontolateral cortex and, to a lesser extent, the orbitofrontal, brainstem and cerebellar submatrix. Selective decreases in connectivity were found for the basal ganglia submatrix, with the frontolateral, motor, somatosensory, insular and brainstem submatrices. The connectivity between the motor and somatosensory submatrices was decreased as well (see Fig. 1B). All reported differences were statistically significant (p < 0.001).

#### Hubs

According to graph-theory, we identified 16 hubs for the HC group and 11 for the PD group, with the anatomical localizations of the hubs differing between groups. In particular, 13 hub regions were lost in PD compared to HC. Lost hubs were part of frontal regions (i.e. inferior frontal gyrus pars opercularis bilaterally and left pars triangularis), the anterior cingulate cortex, the left precentral gyrus, the right angular gyrus, the left insula, the striatum (i.e. left putamen and left ventral striatum) and cerebellum (Fig. 1C).

### Dopamine pathway analysis

**Figure 2:**
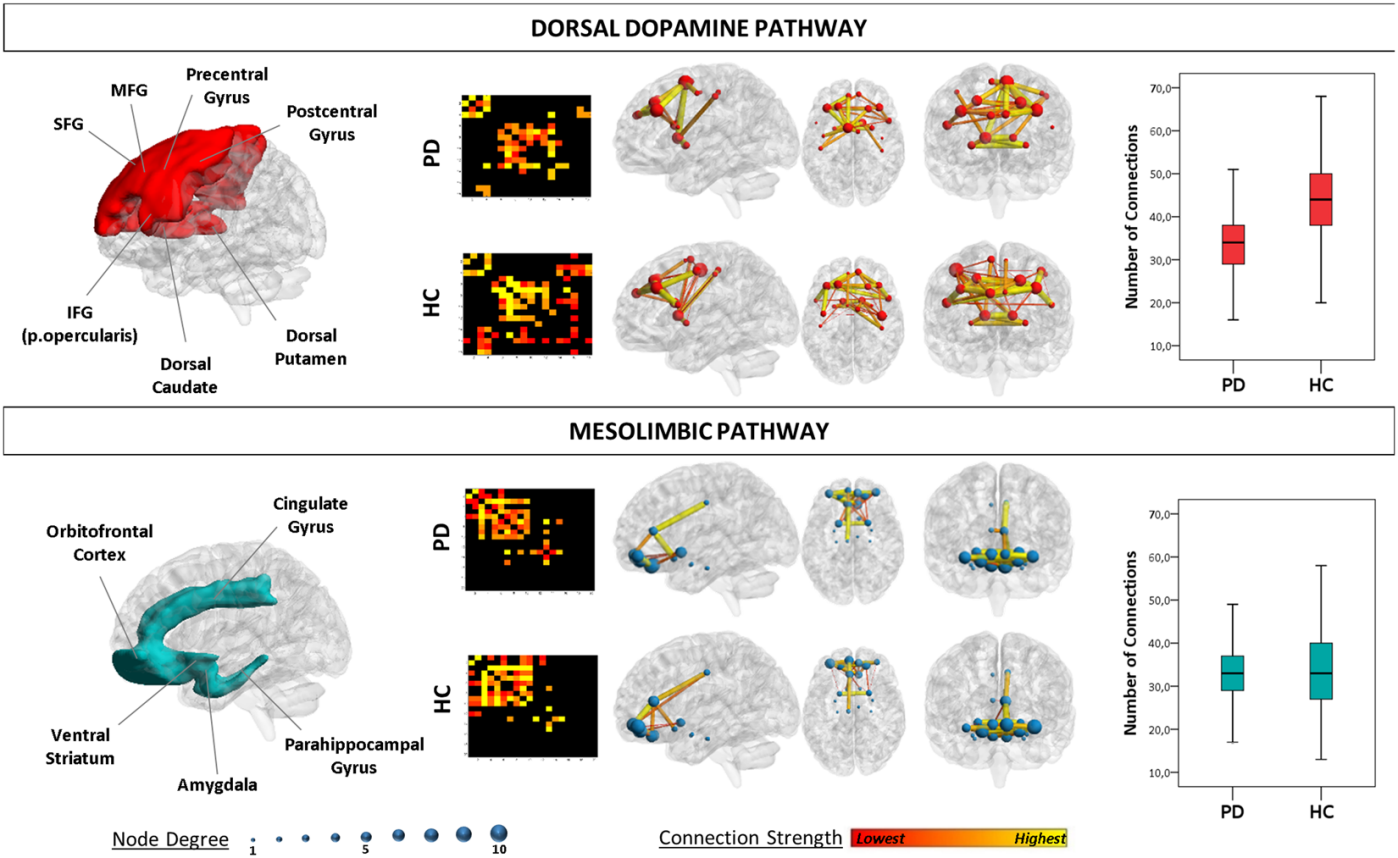
Dopaminergic networks analysis. Figure shows the cortical and subcortical projections of the main dopaminergic pathways (left panel). The dorsal dopamine pathway is affected in PD, showing loss of connectivity and reconfiguration. The mesolimbic pathway is spared, with the overall number of connections in the pathway being preserved. Node degree, i.e. the number of connections for each region, is represented by the size of each dot. Connection strength is represented by the colour and thickness of each line connecting two nodes. Abbreviations: SFG: Superior Frontal Gyrus; MFG: Middle Frontal Gyrus; IFG = Inferior Frontal Gyrus. Box plots (right panel) show the total number of connections in each dopaminergic pathway, for PD and HC. Difference is significant for the dorsal dopamine pathway (p < 0.001).

A significant loss of connectivity in the *dorsal dopamine pathway* was present in PD vs. HC (T = −29.578, p < 0.001). The connections from bilateral putamen, precentral and postcentral gyri, dorsolateral prefrontal cortex, and the right supplementary motor area and superior medial frontal gyrus were affected. The *mesolimbic pathway* was instead globally spared, with no difference in the comparison to HC in the total number of metabolic connections (T = −1.529, p > 0.05) (Fig. 2).

All reported differences were statistically significant (p < 0.001).

### α-synuclein pathology spreading analysis

**Figure 3:**
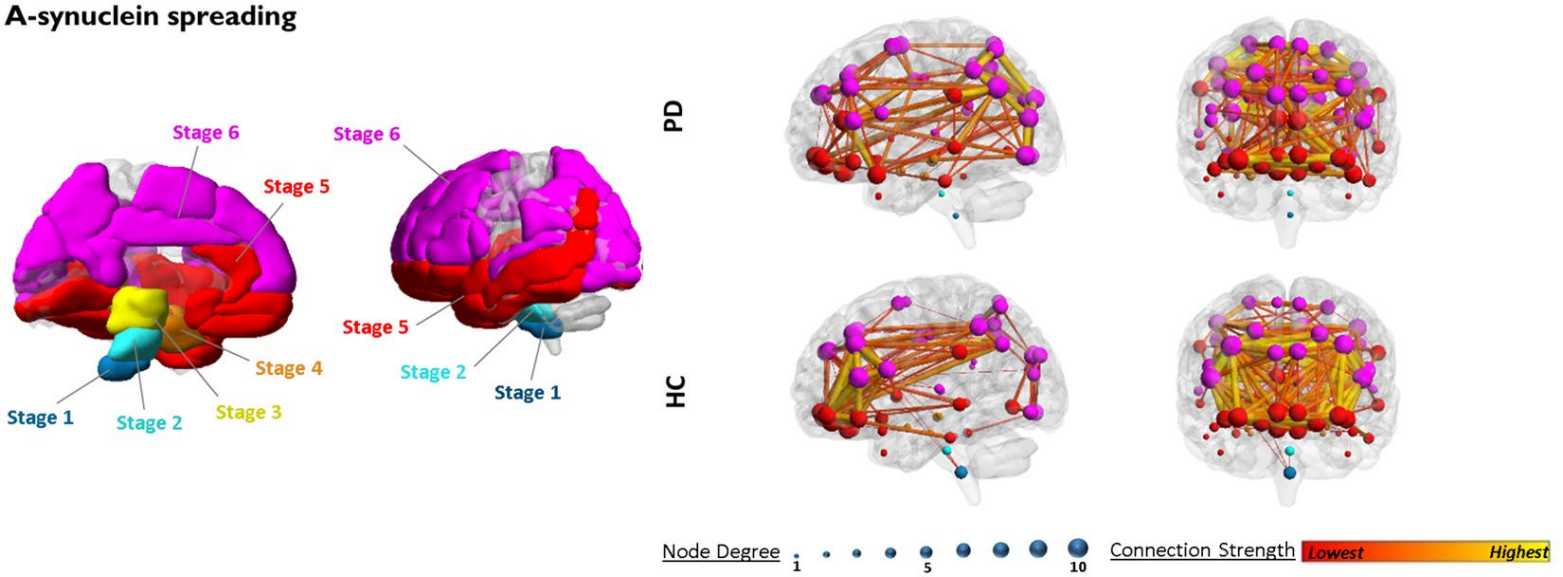
α-synuclein spreading analysis. Figure shows the progression of α-synuclein pathology according to Braak’s staging model (left panel). Metabolic connectivity alterations first appear in the lower brainstem, pons and midbrain (Braak’s stages 1, 2, 3) and furthermore spread to the orbitofrontal regions (stage 5). Connectivity between orbitofrontal (stage 5) and frontolateral cortex (stage 6) was also partly affected (right panel). The ROIs were defined according to AAL atlas (see text for details). α-synuclein-spreading connectivity results projected on a 3D brain template for PD and HC groups (right panel). Node degree, i.e. the number of connections for each region, is represented by the size of each dot. Connection strength is represented by the colour and thickness of each line connecting two nodes.

We found decreased metabolic connectivity between regions early affected by α-synuclein pathology, namely the lower brainstem, pons and midbrain (Fig. 3). Lower brainstem, pons and midbrain also underwent significant metabolic disconnections with the regions later affected by α-synuclein pathology (i.e. belonging to stages 5 and 6). Among regions later affected by pathology, only stage 5 showed significant *local* connectivity derangement (t = −18.415, p < 0.001), particularly affecting the orbitofrontal cortex. Orbitofrontal cortex also suffered *long-distance* disconnections with the frontolateral cortex, a region belonging to the Braak’s stage 6. *Local* connectivity was not affected in Braak’s stage 4 nor 6 (t = 0.199, p > 0.05; t = −0.776, p > 0.05).

### Resting-state networks analysis

**Figure 4:**
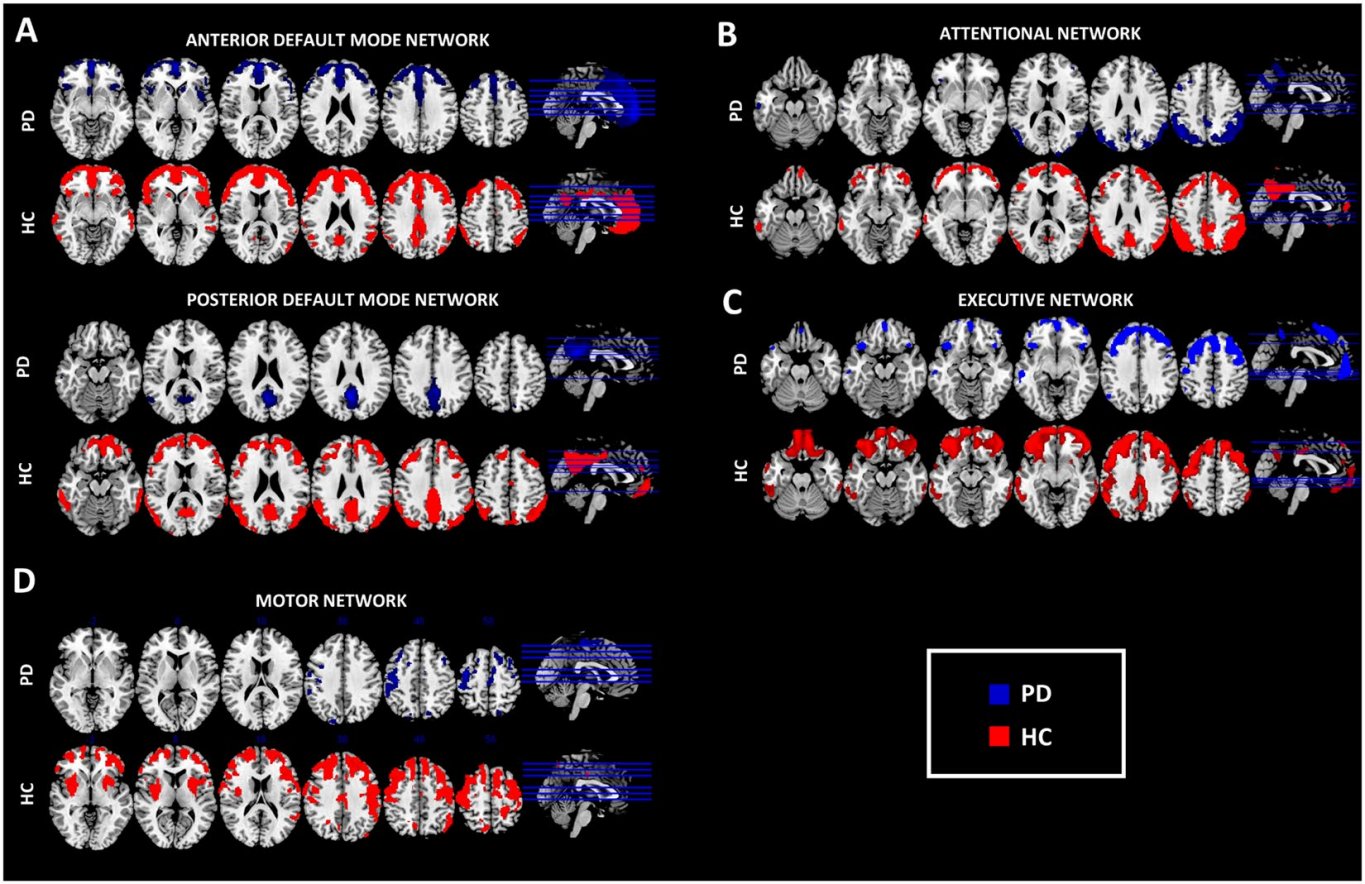
Resting-state networks analysis. Figure shows resting-state networks topography in PD (blue overlaid to the anatomical template) and HC (red overlaid to the anatomical template) for (**A**) anterior and posterior default mode network; (**B**) attentional, (**C**) executive networks, (**D**) motor network. A connectivity derangement is present in all the resting-state systems, particularly in the frontal components of each network. Resting-state networks were obtained using seed-based intercorrelation analysis. Clusters with a minimum spatial extent of 50 voxels are shown, with a voxel-wise significant threshold of p < 0.01 FDR-corrected.

Resting-state networks obtained from the seed-based inter-correlation analysis are shown in Fig. 4. Alterations in the metabolic connectivity maps were present in all the analysed resting-state networks. As for the *attentional network*, a disconnection of the dorsolateral prefrontal cortex from the seed (i.e. the inferior parietal lobule) was observed. Regarding the *posterior DMN*, the anterior regions (i.e. ventromedial, orbital and dorsolateral frontal cortex) were completely disconnected from the seed (i.e. posterior cingulate cortex). The *anterior DMN* showed also loss of connectivity between the seed (i.e. anterior cingulate/ventromedial frontal cortex) and the posterior regions of the network. The *motor network* showed reduced connectivity within the cortical regions (i.e. premotor cortex, superior medial frontal gyrus, dorsolateral prefrontal cortex) and a total disconnection with the subcortical regions (i.e. putamen bilaterally) and from the seed. The *executive network* was affected to a lesser extent, with a loss of connectivity in orbitofrontal, lateral inferior parietal and cingulate regions.

## Discussion

Previous studies investigating metabolic connectivity in PD mainly focused on the identification of specific patterns of metabolic covariance and their association to motor and cognitive symptoms, using principal component analysis^28–30^. Our study applied for the first time an alternative approach, namely SICE in combination with seed-based inter-correlation analysis for a multiscale assessment of metabolic connectivity in PD, by exploiting the unique sensitivity of [18F]FDG-PET in capturing the pathological events affecting brain functionality. Metabolic connectivity, as revealed by [18F]FDG-PET, should reflect in particular the neuropathological events affecting synaptic functions in PD, such as neurodegenerative processes related to aggregation of α–synuclein at the presynapsis^2^, which interferes with synapthophysin^31^ and synapsin-III^32^ proteins functioning, and neurotransmitters alterations^33^.

We assessed whole-brain metabolic connectivity without *a priori* constraints, in order to explore possible local and long-distance disconnections and adaptive compensatory changes in the brain organization. We showed that early PD is associated with a whole-brain architecture reorganization, with connectivity changes largely affecting frontolateral cortex and cerebellum, both locally and at long-distance.

We also assessed pathology-driven alterations in metabolic connectivity by specifically targeting the metabolic architecture of the dopaminergic pathways and the regions involved in α-synuclein pathology spreading. The connectivity within the dorsal dopamine pathway was selectively affected, with concomitant sparing of the mesolimbic pathway. As for the α-synuclein spreading, metabolic connectivity was more impaired in the regions early affected by α-synuclein pathology (Braak’s stages 1–3)^27^.

Finally, we evaluated the degree to which high-order resting state networks are affected in PD, revealing a common pattern of connectivity impairment, with a major disconnection to and within the frontal regions across all resting state networks.

Altogether, these results show that alterations in PD metabolic connectome are strongly consistent with those found in other synucleinopathies such as DLB (cfr. ref. 24), thus supporting a backbone of shared neuronal vulnerabilities consistent with the common underlying pathology.

Connectivity changes at each scale level are discussed in detail as follows.

The **whole brain analysis** revealed *long-distance connectivity decreases* whose most prominent feature was the widespread disconnections affecting the frontolateral submatrix (see Fig. 1). Previous studies in PD have almost exclusively focused on the fronto-striatal circuit^34, 35^, reporting functional disconnections between wide portions of the frontolateral cortex and striatum^34^, with metabolic changes in the dorsolateral and anterior prefrontal regions coupled to caudate dopamine depletion^35^. Cortico-cortical connectivity alterations involving the frontolateral cortex were previously reported in PD, although limited to the connections between frontolateral cortex and premotor/supplementary motor areas^36^. Our results add further evidence for more widespread frontal connectivity changes, showing altered connections with parietal, occipital and temporal cortices, as well as with the basal ganglia, brainstem and cerebellum. The significance of frontal disconnections for specific PD symptoms remains hitherto to be explored. Fronto-striatal system abnormalities, however, are known to be at the basis of PD executive dysfunction, such as planning, spatial working memory and attentional set-shifting^37^. This extensive pattern of connectivity derangement supports the major frontal pathophysiology in early PD^38^.

Cerebellum submatrix showed also widespread long-distance disconnections, particularly with frontolateral and premotor areas. This evidence indicates that also specific cerebellar-cortical networks are involved in PD, together with an abnormal cerebello-thalamic connectivity, which we also found altered^39^.

The basal ganglia submatrix revealed selective connectivity decreases with the frontolateral, premotor, somatosensory, insular and brainstem submatrices, consistent with previous studies using fMRI, that showed a reduced functional connectivity among striatum and dorsolateral prefontal cortex^40^, sensorimotor cortex^41^ and brainstem^9^. The disconnection between basal ganglia and brainstem is particularly relevant, since it is the very early locus of PD neuropathology^27^.

As for the *long-distance connectivity increases*, these were observed in posterior brain regions, namely between the parietal, temporal and occipital cortices. Parietal, occipital and temporal cortices were also the core of a widespread number of connectivity increases involving other cortical and subcortical regions (see Fig. 1). The *cerebellum* showed also selective connectivity increases, that were previously reported using rs-fMRI and ascribed to a compensatory role of cerebellum during the early PD stages^42^. However, the interpretation of connectivity increases as due to compensatory mechanisms needs a note of caution, since evidence suggesting maladaptive mechanisms also exists (cfr. ref. 25). This change in connectivity may indeed reflect ineffective “compensatory” mechanism present in the early PD phase. As all PD patients included in the study were cognitively stable, we might also argue that the presence of compensatory mechanisms acting particularly in the posterior brain regions might contribute to the preservation of cognitive functioning in early PD. A progressive posterior cortical impairment is thought to be related to cognitive decline and development of dementia in PD^43^.

As for *local connectivity decreases*, frontolateral, orbitofrontal and cerebellar submatrices suffered the major loss of local connectivity (see Fig. 1). The frontal lobe dysfunction is an established feature of PD^38^, and considered a major consequence of the striatal dopaminergic deficits, running through the strong interconnections between frontal cortex and striatal regions^38^. Consistently, reductions in glucose metabolism in both prefrontal and orbitofrontal regions have been reported in PD and associated with dopaminergic striatal depletion^35, 44^. Intra-cortical dopamine deficits have been suggested to play a role in the regulation of prefrontal activity in PD, with dopamine depletion leading to defective activation of the dorsolateral prefrontal cortex during motor tasks^45^.

As for the cerebellum local loss of connectivity, we might argue that a crucial role might be played by pathological changes such as α-synuclein pathology^46^, dopaminergic degeneration^42^, loss of dopaminergic D_1_-D_3_ receptors^47^, atrophy^48^, and white matter damage^49^. Cerebellum is involved in the planning and execution of motor movements, postural control and tremor as well as in a wide range of cognitive functions^39^ and its derangement, as clearly shown in the present paper, crucially highlights its role in PD.

We found *local connectivity increases* in cortical temporal, parietal and occipital regions that were previously interpreted as compensatory mechanisms, occurring in the extra-striatal regions^50, 51^ (see Fig. 1). In addition, we found local striatal connectivity increases, as also previously reported^40, 41^. Compensatory mechanisms within the striatum have been suggested to play a role in particular during the very early disease phases, with extra-striatal compensatory mechanisms acquiring importance with disease progression^50^. A recent study has shown that striatal compensatory mechanisms are pivotal to compensate for striatal dopaminergic depletion and maintaining a normal cognitive functioning^52^.

As for the **dopaminergic systems analysis**, the results notably showed a different vulnerability of the two dopaminergic systems, with a significant loss of connectivity in the dorsal dopamine pathway, and a preserved connectivity in the mesolimbic pathway (see Fig. 2). Dopamine depletion impairs the frontostriatal functional connectivity contributing to the pattern of altered metabolic connectivity here observed^53^. In addition, α-synuclein plays a key role in both dopaminergic neurotransmission pathways, i.e. regulating the filling and fusion of vesicles via vesicular monoamine transporter 2 and the dopamine reuptake via dopamine transporter^2^. It has been shown that substantia nigra neurons are more vulnerable to α-synuclein pathology than ventral tegmental area neurons, possibly due to the higher energy demand of the former^54^. The present findings confirm for the first time at the “metabolic level” the specific dorsal dopamine pathway involvement in early PD patients. Comparable findings were also reported in our previous work on metabolic connectivity in DLB, thus showing that a major vulnerability of the dorsal dopamine pathway is not a disease-specific feature of PD, but extends to other diseases in the synucleinopathies spectrum^24^.

The pattern analysis of **α-synuclein pathology spreading** revealed altered metabolic connectivity in the regions early affected by α-synuclein pathology (see Fig. 3). The specific connectivity impairment involving the regions of interest (ROIs) part of the first three Braak’s stages (i.e. midbrain, pons and lower brainstem) is consistent with the pathology model postulating that the clinical signs of parkinsonism appear when α-synuclein pathology reaches the third stage, and degeneration of substantia nigra occurs^27^. Notably, these regions were also disconnected at long-distance from regions later affected by α-synuclein pathology, ﻿﻿i.e﻿. the regions belonging to stage 5 and 6.

We also found a partial local connectivity impairment in stage 5 (i.e. the orbitofrontal cortex) and in stage 6 (i.e. the frontolateral cortex). The orbitofrontal and frontal lateral cortex have consistently been reported as loci of damage in PD^49, 55^, due to the dopamine-mediated striato-frontal disconnection^35, 38, 44^. Stage 4 did not show significant local connectivity changes. From the methodological standpoint, it must be acknowledged that the anatomically-restricted analyses for the α-synuclein spreading were performed using the automated anatomical labelling atlas (AAL) functional-anatomical regions, not specifically constructed for pathology-driven analysis. Stage 4, that includes temporal mesocortex and allocortex, might have suffered for the ROIs subdivision derived from the AAL. In the future, atlases based on histopathology may become available, allowing this limitation to be overcome. Alternatively, it is possible that the “hippocampus, parahippocampus and amygdala” ROIs we chose for Stage 4, could be less affected by α-synucleopathy in PD, given the complete absence of memory related deficits in our series. Taken together, these results support the link between α-synuclein accumulation and synaptic dysfunction as measured by [18F]FDG-PET and notably give an *in vivo* support to Braak’s staging model^27^. Also, this result is consistent with our previous α-synuclein-spreading-based connectomics analysis in DLB^24^, thus *in vivo* supporting a common pattern of α-synuclein-spreading across the two diseases belonging to the same pathology spectrum.

As for **resting-state networks analysis**, we found a loss of connectivity in the dorsolateral prefrontal cortex and the parietal regions, affecting the attentional network (see Fig. 4B), consistently with a previous rs-fMRI study in PD^11^, which reported also an association between the impairment of attention network hubs and poor performance in perceptual tasks.

As for *DMN*, there was a loss of connectivity in both the anterior and posterior components (see Fig. 4A). This pattern of disconnection has been previously described in normal aging and, at an accelerated rate, in Alzheimer’s disease^56^. The author speculated that this pattern of disconnections could be due to age-related increasing synaptic inefficiency, to which subject vulnerable to develop Alzheimer’s disease would be more susceptible^56^. Our results suggest that not only Alzheimer’s disease but also PD would produce a similar pattern of connectivity impairment in the DMNs that might be prodromal to further development of cognitive symptoms. Notably, recent studies have shown that DMN is disrupted even in cognitively unimpaired PD patients^10, 57^. It has been suggested that DMN disruption mainly arises as a direct consequence of PD pathology^57^ and maximally as the result of PD dopaminergic impairment^58^. Consistently, it was shown that administration of drugs enhancing dopaminergic activity is able to restore normal DMN functioning in PD^58^.

As for the *executive network*, our results strengthen the link between the dysfunction of the executive network and the alteration of frontal-striatal connectivity (see Fig. 4C). Previous rs-fMRI studies in PD also reported abnormal connectivity within this network^59^, as well as abnormal interactions between the executive network and DMN^60^. The impairment in prefrontal and orbitofrontal regions, considered to be at the basis of the executive dysfunctions in PD, might follow frontal-striatal dopaminergic imbalance^61^. Notwithstanding the role of frontal impairment in the executive dysfunction is well-recognized in PD, it is not clear whether it contributes to cognitive decline and dementia development^62^. An early predictor of dementia in PD is instead the posterior cortical damage^62^. The dual syndrome hypothesis considers the fronto-striatal dysfunction as a characteristic of non-demented PD patients, and the posterior cortical and temporal lobe dysfunction as a signature of rapid cognitive decline^63^.

This study, together with our previous work in DLB^24^, allows for a comparative picture of the metabolic connectome in the two most diffuse α-synucleinopathies. Notably, the whole-brain analyses in PD and DLB groups revealed consistencies and substantial differences. As for the differences, PD showed an extensive decrease of connectivity in frontal regions, with compensatory connectivity increases involving occipital and posterior cortical regions, whereas an occipital connectivity impairment was present in DLB, together with frontal compensatory connectivity increases. This opposite pattern of anterior-posterior connectivity changes in early cognitive stable PD and in DLB patients might be of crucial clinical relevance. It is consistent with the prevalent frontal functional changes previously reported in non-demented PD patients, as opposed to the posterior impairment associated with dementia in PD and DLB. The different impairment of anterior-posterior connectivity might reflect the different vulnerability of the cholinergic system in PD and DLB^61^, leading to disease-specific patterns of neuronal dysfunction^43, 64–66^, and metabolic connectivity changes, here reported for the first time.

As for the consistencies, cerebellum, mesencephalic-pontine and striatal regions showed comparable patterns of altered connectivity in both PD and DLB, locally and at long-distance. Pathology-driven analyses provided similar results in the two parkinsonian conditions, with the greatest connectivity impairment in the dorsal dopamine pathway and in the regions early affected by α-synuclein pathology. Further studies within this spectrum, involving populations of PD patients with mild cognitive impairment or dementia, DLB patients at different disease phase and MSA cases, will provide the evidence for common and disease-specific connectivity alterations also linked to the specific clinical phenotypes.

In conclusion, our results show that metabolic connectivity is affected in early PD at multiple scale levels, with (i) a thorough reconfiguration of the global whole-brain architecture, mainly affecting frontal connectivity, (ii) a metabolic connectivity disruption underlying a major impairment in the dorsal dopamine neurotransmission system and in the early spreading of α-synuclein pathology, and (iii) reconfigurations in high-order resting state networks affecting in particular the frontal regions. The reported results point at a disease-specific pattern of frontal disconnection and posterior connectivity compensation, consistent with the “*dual syndrome hypothesis*”^63^. The present results support the emerging hypothesis that PD is a widespread neurodegenerative disease at multiple scale levels (cfr. ref. 6) and that the era of the axiom “PD equals dopaminergic dysregulation” needs to be rewritten.

## Materials and Methods

### Subjects

Thirty-four idiopathic PD patients without any cognitive impairment at baseline and stable during 4-years follow-up were retrospectively collected from the clinical and imaging database of the Neurology Unit, Department of Clinical and Experimental Sciences, at the University of Brescia, Brescia, Italy. The clinical diagnosis was made according to the UK Brain Bank Criteria^67^ by movement disorders specialist (A.P. and A.A.), considering full medical history, neurological examination (including UPDRS-III in ON condition) and a standard neuropsychological assessment. All patients included underwent structural imaging (magnetic resonance imaging or computed tomography (CT)) in order to exclude prominent cortical or subcortical infarcts or brain/iron accumulation or atypical parkinsonian disorders. Stringent exclusion criteria were applied: presence of deep brain stimulation, verified genetic mutation known to cause PD, concomitant psychiatric or other neurological disorder, hallucination, psychosis or antipsychotic drug use, history of drug or alcohol abuse, mild cognitive impairment. The study crucially excluded PD patients with a mild cognitive impairment or dementia diagnosis defined according to the level II MDS criteria using the neuropsychological assessment and cut-offs recently published^68^. The PD patients included underwent [18F]FDG-PET scans for research purposes. The group comprised 18 males and 16 females with a mean age of 63.71 ± 11.70 years and mean disease duration of 4.5 ± 2.65 years. No patients included in the study developed dementia at 4-year clinical and neuropsychological follow-up, thus presenting a stable cognitive functioning at least until 8.5 ± 2.65 years of disease duration. All patients were taking antiparkinsonian medications and had a mean Levodopa equivalent daily dose of 422 ± 316 mg. Demographic, clinical and cognitive features in PD and healthy controls (HC) groups are reported in Table 1.

**Table 1:** Demographic, clinical and cognitive features of the study groups.

	PD	HC	*p*
N	34	34	—
Gender (M/F)	18/16	17/17	0.808
Age (years ± sd)	63.71 ± 11.70	65.55 ± 8.85	0.667
Disease Duration (years ± sd)	4.5 ± 2.65	—	—
UPDRS-III (ON)	14.7 ± 1.2	—	—
Hoehn & Yahr staging, N (%)			
1	9 (26%)	—	—
2	15 (44%)	—	—
3	10 (30%)	—	—
4	—	—	—
MMSE	28.61 ± 0.28	—	—
Total LEDD (mg/die ± sd)	422 ± 316	—	—

**Abbreviations:** HC, healthy controls; LEDD, Levodopa equivalent daily dose; MMSE, Mini-Mental State Examination; PD, Parkinson’s disease patients; UPDRS-III, Unified Parkinson’s Disease Rating Scale- motor score.

[18]FDG-PET scans of 34 age-matched cognitively normal HC were included for comparison. The group comprised 17 males and 17 females with a mean age of 65.55 ± 8.85 years. Age and gender did not differ significantly between PD and HC groups (Age: T = 0.433, p = 0.667; Gender: Χ^2^ = 0.059, p = 0.808).

All subjects or their informants/caregivers gave their written informed consent to the experimental procedure, as approved by the Ethical Committees of Spedali Civili di Brescia and of San Raffaele Hospital. The protocol conformed to the ethical standards of the Declaration of Helsinki for protection of human subjects.

### [18F]FDG-PET image acquisition and pre-processing

The [18F]FDG-PET scans were acquired using a Discovery STE PET scanner (3.27 mm thickness; 5.55 mm in-plane full width at half maximum (FWHM)), manufactured by GE Healthcare. The [18F]FDG-PET acquisition procedures conformed to the European Association of Nuclear Medicine guidelines^69^. Static emission images were acquired 45 min after injecting 185–250 MBq of [18F]FDG via a venous cannula, with scan duration of 15 min.

Uniform reconstruction protocols were applied in order to factor out possible sources of variability. All images were reconstructed using an ordered subset-expectation maximization algorithm (OSEM). Attenuation correction was based on CT scans. Each reconstructed image was visually inspected in order to check for major artefacts, i.e. defective image uniformity/orientation or attenuation correction due to mismatch between CT and PET images.

Images underwent general pre-processing procedures, using the MATLAB (Mathworks Inc., Sherborn, Mass., USA) based software SPM5 (http://www.fil.ion.ucl.ac.uk/spm/software/SPM5/). Each image was first normalized to a [18F]FDG-PET specific template registered to the MNI standard space^70^ and spatially smoothed with an 8 mm isotropic 3D Gaussian FWHM kernel. Images were then proportionally scaled to the global mean.

### Whole-brain and pathology-driven metabolic connectivity analyses

We used **SICE**, a method previously validated by Huang *et al*.^19^. SICE-based analyses involve the definition of the basic units of each network, i.e. the ROIs/nodes. The [18F]FDG-PET signal is extracted from each ROI and from each subject, thus obtaining SubjectsxROIs matrices. SICE algorithm is then applied on the SubjectsxROIs matrices in order to estimate the metabolic connectivity matrices. Graph theory measures are computed from the metabolic connectivity matrices and a bootstrapping procedure is performed in order to test differences between HC and PD groups.

#### Node selection and ROIs definition

For the *whole-brain*, we created the whole-brain metabolic connectivity matrix (i.e. the full-matrix), considering cortical, subcortical, cerebellar, and brainstem regions(121 × 121 ROIs). Following Huang and colleagues (2010), the full-matrix was subdivided into 13 sub-matrices, representative of larger brain regions^19^. We considered frontolateral, fronto-orbital, frontal medial, motor, somatosensory, parietal, occipital, temporal, insula, thalamus, basal ganglia, brainstem and cerebellar submatrices for statistical testing (see Fig. 3B).

In the pathology-driven connectivity analyses, we focused on the neural networks related to PD pathology, namely the dopaminergic neurotransmission pathways^26^ and the brain regions affected by the spreading of α-synuclein according to Braak’s staging^27^.

For the *dopaminergic system* *analysis*, we considered ROIs pertaining to the dorsal and the mesolimbic dopamine pathways, as detailed elsewhere^24^. The small dopaminergic nuclei, i.e. substantia nigra and ventrotegmental area, were not included in the analysis, due to the limited spatial resolution of PET method and the lack of reference atlases for these regions.

As for the *α-synuclein spreading analysis*, we considered the staging system proposed by Braak and colleagues (2003)^27^. Within the α-synuclein network, we considered the lower brainstem and pons for stages 1 and 2 respectively, since these regions contain the grey matter nuclei early affected by α-synuclein pathology in PD, i.e. the dorsal IX-X motor nuclei, intermediate reticular zone, caudal raphe nuclei, gigantocellular reticular nucleus and coeruleus/subcoeruleus complex; the midbrain, which includes the pathological hallmark of the disease, namely the substantia nigra *pars compacta* for stage 3; hippocampus, parahippocampal gyrus, and amygdala for stage 4; the temporal lobe, anterior cingulate, insular and orbitofrontal cortices for stage 5; the remaining neocortical areas, with the exception of the primary sensory areas and the primary motor fields, for stage 6^27^ (Fig. 3).

All the above mentioned ROIs were derived from the same atlas, i.e. AAL^71^, with the exception of ROIs not included in the AAL, namely (i) the brainstem ROIs, (midbrain, pons, lower brainstem) derived from the WFUPickAtlas Talairach Daemon Lobar Atlas^72^; (ii) the dorsal and ventral striatum ROIs defined considering the basal ganglia boundaries as provided by the AAL and the subdivision provided by the Tziortzi and colleagues (2011) guidelines^73^.

#### Construction of Brain Connectivity Matrices

Estimation of Brain Connectivity Matrices was based on SICE, a method suitable for metabolic connectivity studies, where the traditional maximum likelihood estimation method cannot be reliably applied^19^. Construction of Brain Connectivity Matrices was performed using GraphVar toolbox^74^. SICE allows to estimate the structure of the metabolic connectivity matrix by imposing a sparsity constraint on the maximum likelihood estimate of the inverse covariance matrix^19^. SICE provides a robust estimation of the binary structure of the inverse covariance matrix, but it does not allow to estimate the strength of non-zero entries due to shrinkage^23^. As a consequence, only information on the presence or absence of metabolic connections can be used to build the brain connectivity model^19^. Though information on the strength of the metabolic connections are not provided by SICE, a quasi-measure of strength can be obtained by summing up unweighted binary matrices estimated at different density levels^19^. Only for visualization purposes, we used Stability Approach to Regularization Selection (StARS) (see below) applied to matrices at a predefined range of densities (i.e. 3–18% with an incremental interval of 3%), in order to obtain unweighted binary matrices at different densities. By summing up the six unweighted binary matrices for each group and analysis, summary quasi-weighted matrices were obtained and represented in a 3D brain template using BrainNet toolbox^75^.

Given that robustness of the sparse estimation depends on matrix density^76^, density level must be carefully chosen to ensure that the covariance matrix is sparse^19^. In SICE, density level is controlled for by a regularization parameter λ^19^. Different strategies have been proposed for density selection^77^ but no gold standard was established for selection of λ using SICE. A new criterion for λ selection, called StARS, has been recently proposed^78^. StARS allows to select the minimum value of λ necessary to ensure matrix sparsity and replicability under random sampling, avoiding the risk of massive false positives without failing to capture the true structure of the connectivity matrix^78^. In order to select the optimal sparsity level for each group, StARS^78^ was applied to both PD and HC matrices. The resulting matrices were used for statistical testing.

#### Graph theory and statistical analyses

For the whole-brain analysis, we computed the degree and the participation coefficient of each node using Brain Connectivity Toolbox^76^. Participation coefficient (Pi) was defined as:

$${P}_{i}=1-\sum _{m=1}^{Nm}{(\frac{{k}_{i}(m)}{{k}_{i}})}^{2}$$

where N_m_ is the number of modules, k_i_(m) is the number of links of node *i* to nodes in module *m*, and k_i_ is the total degree of node *i* ^79^ Partecipation coefficient was adopted in order to identify the most important regions/nodes of the network, i.e. the hubs^80^ by setting a threshold at 1.25 standard deviation over the average participation coefficient.

Considering the whole-brain sub-matrix-based analysis, we calculated the total number of within or local connections (i.e. connections linking two nodes in the same sub-matrix) and the total number of between or long-distance connections (i.e. connections linking two nodes belonging to different sub-matrices). For the pathology-driven networks, we computed the total number of connections and nodal degree within each network.

A bootstrapping procedure was adopted in order to rigorously test differences between groups^19^. For each connectivity matrix, we extracted 1000 bootstrap samples of 34 subjects, with replacement, for both PD and HC groups. Based on PD and HC respective bootstrap samples, we performed hypothesis testing for each SICE analysis. For the whole-brain analysis, we tested significant differences between PD and HC groups in the total number of connections within each submatrix and between each pair of submatrices. For the pathology-driven analysis, we tested the null hypothesis that the total number of connections in each dopaminergic pathway and in each Braak’s stage was equal across groups. Multiple independent t-tests with Bonferroni correction were performed for each analysis. For a thorough explanation of the statistical testing procedure see ref. 19.

### Resting-state networks metabolic connectivity

We used **seed-based inter-correlation analysis**, a method previously validate by Lee and colleagues (2008) for [18F]FDG-PET data^20^. We focused our analyses on the attentional, DMN (anterior and posterior), executive and motor networks. For each network, a seed region was selected on the basis of previous literature, namely the inferior parietal lobule for the *attentional network* ^81^, the anterior cingulate cortex/ventromedial prefrontal cortex for the *anterior* DMN^56^, the posterior cingulate cortex for the *posterior DMN* ^82^, the dorsolateral prefrontal cortex for the *executive network* ^83^, and the precentral gyrus for the *motor network* ^84^. For each group and network, [18F]FDG-PET signal extracted from the designated seed was entered as covariate in a Multiple Regression Model in SPM5 and used to predict [18F]FDG-PET signal elsewhere in the brain, thus allowing the estimation of the corresponding resting state network. Voxel-wise False Discovery Rate (FDR)-corrected p < 0.01 was considered statistically significant.

### Data Availability

All relevant data are included in this published article.
